# Complete Resection of Giant and Well-Differentiated Retroperitoneal Liposarcoma: A Case Report

**DOI:** 10.7759/cureus.89927

**Published:** 2025-08-12

**Authors:** Lei Zhang, Jiawei Zhang, Bo Chen, Yan Li, Zhengyu Hu

**Affiliations:** 1 Department of Gastrointestinal and Hernia Surgery, The First Affiliated Hospital of Anhui Medical University, Hefei, CHN; 2 Core Facility Center, The First Affiliated Hospital of the University of Science and Technology of China (USTC), Hefei, CHN

**Keywords:** giant abdominal tumor, retroperitoneal liposarcoma, surgery, three dimensional reconstruction, well differentiated liposarcoma

## Abstract

Retroperitoneal liposarcoma is a rare soft-tissue malignancy that occurs in middle-aged to older males. In the present case, the tumor was so large that it occupied almost the entire abdominal cavity. With the help of a three-dimensional reconstruction technique, we completely resected the tumor, and the pathology showed that it was a well-differentiated sarcoma, and the tumor did not recur after one year of postoperative follow-up. We present the clinical, radiographic, and pathological findings of such a case.

## Introduction

Liposarcomas are rare malignant tumors arising from the differentiation of adipocytes. Retroperitoneal liposarcoma (RPLS) is the most common retroperitoneal soft tissue tumor for which surgery is the fundamental treatment [1]. The incidence rate is roughly the same for males and females, with the peak incidence occurring between the ages of 60 and 70. The initial symptoms and signs are nonspecific and include increased abdominal girth, early satiety, and gastrointestinal obstruction. CT and MRI show that the tumor consists mainly of fatty tissue. Here, we present a 58-year-old male patient with well-differentiated giant retroperitoneal liposarcoma. The treatment we reported may contribute to improving the prognosis.

## Case presentation

**Figure 1:**
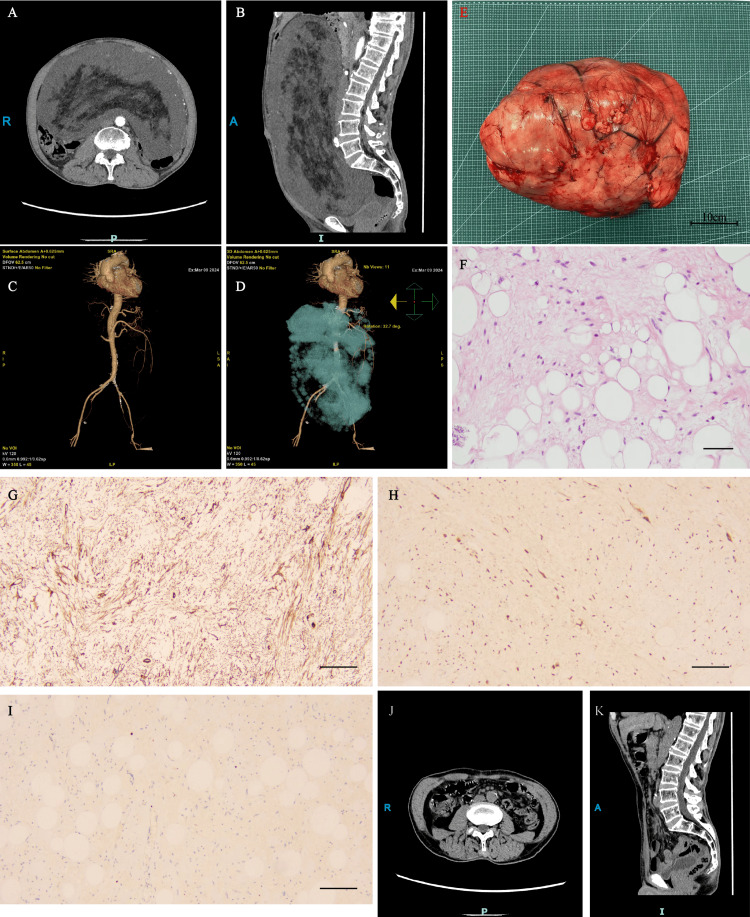
Imaging and pathological examination images of the patient (A, B) Abdominal CT showed a retroperitoneal space-occupying lesion. On enhanced scanning, solid nodules showed moderate progressive enhancement. Polymorphic undifferentiated liposarcoma was considered. (C, D) 3D reconstruction showing the spatial relationship between the tumor and major blood vessels. (E, F) The tumor volume was about 44.7*29.5*12.8 cm, weighing 12.6 kg, and was confirmed as a liposarcoma by HE staining. (G-I) Immunohistochemical staining results: vimentin (+), MDM2 (+), KI-67(+, Index:1%). (J, K) CT examination one year after surgery.

A 58-year-old male patient presented to the clinic with persistent fullness and discomfort after eating for the past month, with no previous symptoms. CT examination showed a large tumor occupying the abdominal cavity, extending from the level of the raphe to the pelvis, with liquefied necrosis in the center, which was considered to be a liposarcoma. Preoperatively, we performed a three-dimensional reconstruction of the tumor and vessels to assess the relationship between the superior mesenteric vessels and the tumor. After ruling out contraindications to surgery, we made a 20 cm median incision, ligated the main trophoblastic vessels of the tumor, and completely resected the tumor from the mesenteric root. The tumor volume was about 44.7*29.5*12.8 cm and weighed 12.6 kg (Figure 1). It was confirmed that there was no damage to the mesenteric vessels and ureter, with no tumor remnants, and the operation was completed.

Fortunately, postoperative immunohistochemical staining results: CK (-), S-100 (-), desmin (-), P53 (-), ALK (-), vimentin (+), CDK4 (+), MDM2 (+), Ki-67 (+, 1%), pathological diagnosis was well differentiated liposarcoma (WDL), One year after surgery, a follow-up CT examination showed no signs of tumor recurrence (Figure 1).

## Discussion

Liposarcoma typically occurs in middle-aged to older adults, particularly within the retroperitoneum or extremities. Due to the absence of bony boundaries in the retroperitoneal space, liposarcoma can grow extensively in the retroperitoneal space. Clinical symptoms such as abdominal discomfort, weight loss, and abdominal mass usually indicate advanced tumor growth and involvement of surrounding structures, which makes surgery more difficult. The degree of malignancy of RPLS is often determined by the type of pathologic differentiation. Liposarcomas can be divided into four types: poorly differentiated, pleomorphic, myxoid/round cell type, and well-differentiated. The poorly differentiated and pleomorphic types are highly malignant and aggressive, whereas the myxoid/round cell type is low to moderately malignant. The well-differentiated subtypes have a good prognosis with five-year survival rates ranging from 83% to 90% [2,3].

Complete surgical resection is the mainstay of treatment for retroperitoneal liposarcoma, as the efficacy of chemotherapy and radiotherapy remains controversial [4,5]. Surgery for retroperitoneal liposarcoma often requires combined multi-organ resection due to its growth pattern and blood supply characteristics. Preoperative three-dimensional reconstruction CT can show the relationship between the tumor and the surrounding organs and its main blood supply source vessels in three dimensions. In this case, three-dimensional reconstruction CT showed that the main blood supply of the tumor originated from the superior mesenteric vessels, so during the operation, we cut off the tumor blood vessels as much as possible in the secondary vascular arch, which ensured sufficient blood supply to the small intestine and avoided necrosis of the small intestine.

## Conclusions

Although RPLS is rare, based on the Chinese population base, CT scanning is recommended for patients with unexplained abdominal masses, bloating, or abdominal discomfort, as early identification is critical for surgical intervention. Meanwhile, local recurrence is the main cause of death in patients with RPLS, so close follow-up is essential. The risk of recurrence is highest in the first few years after surgery. However, we recommend longer-term follow-up because there have been case reports of RPLS recurrence after more than five years. Regular CT scans are recommended to monitor recurrence. The complexity of RPLS is not limited to the surgical operation itself; ongoing and long-term follow-up is equally critical. Regular monitoring is essential for early detection of any potential recurrence, as tumor recurrence often brings a significant challenge for subsequent treatment.
